# Depressive symptoms hopelessness self-efficacy and COVID-19-related stressors among pregnant women in Hungary across two pandemic waves

**DOI:** 10.1038/s41598-026-57439-9

**Published:** 2026-06-12

**Authors:** Renáta Kovács-Berta, Fanni Dudok, Szabolcs Várbíró, Norbert Pásztor, Edina Dombi

**Affiliations:** 1 Albert Szent-Györgyi Medical School, Doctoral School of Clinical Medicine, University of Szeged, Szeged, 6721 Hungary; 2 Juhász Gyula Faculty of Education, University of Szeged, Hattyas Utca 10, Szeged, 6725 Hungary; 3 Department of Obstetrics and Gynaecology, University of Szeged, Szeged, 6725 Hungary

**Keywords:** Pregnancy, COVID-19-related stressors, Depressive symptoms, Hopelessness, Perinatal mental health, Self-efficacy, Anxiety, Depression

## Abstract

Depression and hopelessness during pregnancy may be intensified by public health crises, yet less is known about how pregnancy- and pandemic-related stressors differ across successive COVID-19 waves. This study examined depressive symptoms, hopelessness, self-efficacy, and COVID-19-related concerns among pregnant women in Hungary during two independent cross-sectional pandemic waves. A total of 692 pregnant women participated in Wave 1 and 459 in Wave 2. Participants completed the Edinburgh Postnatal Depression Scale, Beck Hopelessness Scale, General Self-Efficacy Scale, and the study-specific „Pregnancy during the coronavirus” questionnaire. EPDS categories did not differ significantly between waves; 11.8% of participants in Wave 1 and 10.5% in Wave 2 fell into the major depressive symptom range. Mild hopelessness was reported by 31.1% and 26.4%, and moderate/severe hopelessness by 3.2% and 3.3%, respectively. Confinement-related difficulties and daily disruption, as well as perceived support and positive reappraisal, were higher in Wave 1 than Wave 2, both p < 0.001. In Wave 2, unplanned or unwanted pregnancy was associated with higher depressive symptoms, hopelessness, and financial/housing concerns. Emotional distress and impaired coping was the strongest predictor of EPDS and BHS scores, while self-efficacy was a consistent negative predictor. These findings support pregnancy-sensitive mental health screening during public health crises.

## Introduction

In 2020, the World Health Organization declared the coronavirus disease 2019 (COVID-19) outbreak a global pandemic^1^. Public health emergencies and other accidental crises, including natural disasters, political instability, and economic hardship can trigger sustained stress responses and may contribute to adverse maternal outcomes. Pregnant women may be particularly vulnerable to such stressor, as pregnancy is accompained by substantial physiological, psychological, and social changes that may increase susceptibility to stress-related health problems^1–3^. During public health emergencies, additional stressors such as quarantine, social distancing, reduced in-person healthcare contact, transition to telemedicine, and difficulties in accessing antenatal and postpartum care may further increase psychological burden^4^. In this context, supportive social relationships may play an important role in emotional regulation, stress coping, resilience, and subjective well-being^1,5,6^.

COVID-19-related public health measures substantially altered perinatal care and birth experiences, often by limiting access to interpersonal and professional support. Restrictions on hospital visits and the presence of support person affected many women’s expectations and experiences of childbirth-related care^7^, despite the well-documented benefits of continuous support during childbirth^8,9^. Policies intended to reduce infection risk, including temporary separation of newborns from mothers in some settings, were associated with increased postpartum anxiety and depressive symptoms^10,11^. Pandemic-related stress may also have implications for maternal–fetal bonding, which is considered an important precursor of postnatal mother–infant relationship quality^12–16^, and has been associated with delayed infant motor and communication development^17^. In addition, chronic stress may elevate cortisol levels, with potential implications for fetal development, preterm birth risk, and maternal mental health^3,18^. Traumatic birth experiences may further disrupt maternal bonding and couple dynamics^19,20^.

The pandemic also highlighted the close relationship between mental and physical health. Chronic stress and anxiety during the pandemic have been associated with more severe COVID-19 symptoms and prolonged recovery, and Mendelian randomization evidence has suggested that anxiety may increase the risk of severe COVID-19 outcomes^21^. Among pregnant women, a systematic review and meta-analysis reported substantial psychological burden during the pandemic, with 24.9% of women experiencing depressive symptoms, 32.8% anxiety, 29.4% stress, and 27.9% post-traumatic stress symptoms^22^. These difficulties have been linked to fear of infection, disruptions in prenatal care, uncertainty about the future, concerns about pregnancy complications, and worries about the possible effects of infection on fetal development^7,22^.

Early pandemic studies further suggests that depressive and anxiety symptoms were elevated among pregnant women during the COVID-19 outbreak. In China, the prevalence of depressive symptoms among pregnant women increased significantly during the early phase of the pandemic, from 26.0% to 29.6%^23^. Canadian studies similarly reported substantial psychological distress in this population, with depressive symptoms affecting 37–57% of pregnant women and anxiety symptoms reported by up to 57%; insomnia was also frequently observed^24,25^. These emotional states during pregnancy may affect breastfeeding attitudes, mother-infant interaction, and sexual functioning^26–28^. In a Swedish mixed-methods study of 1577 pregnant women across the first two COVID-19 waves, depressive symptom screening rates were 18.3% in the first wave and 20.1% in the second, while anxiety was significantly more prevalent during the first wave. Difficult social isolation was reported by approximately 40% of participants and was associated with higher depressive symptom prevalence across both waves^29^. In a Swiss online survey, Favre et al. found that 32.9% of pregnant individuals reported severe depressive symptoms and 37.0% met criteria for mental health impairment. Using a specifically designed nine-item COVID-19 concern questionnaire developed from clinical experience and patient feedback, they showed that pandemic-related worries about pregnancy, fetal health, healthcare information, media exposure, and family concerns were relevant to pregnant individuals’ mental health^30^.

In Hungary, the first and second waves of the COVID-19 pandemic represented distinct epidemiological and social contexts. On 11 March 2020, the Hungarian Government declared a state of emergency across the country to mitigate the consequences of the epidemic and protect citizens’ health and lives. The first wave was characterized by rapidly introduced restrictive measures, including visitor bans in healthcare institutions, restrictions on mobility and social contact, and the transition of education to digital formats^31^. By contrast, the second wave was marked by a substantially increased epidemiological burden and the reintroduction of restrictive measures, including night-time curfews, restrictions on gatherings, and limitations on hospitality services^32^. Based on the relevant Hungarian governmental decrees, the two waves may therefore have represented not only different time periods^31,32^, but also distinct stress contexts for pregnant women in Hungary.

Although previous Hungarian research examined pregnant women’s self-rated health status, health behaviour, and hygiene practices during the COVID-19 pandemic^33^, mental health outcomes among pregnant women in Hungary during the pandemic remain insufficiently studies. To our knowledge, no previous Hungarian study has assessed mental health indicators among pregnant women using data collected during separate COVID-19 pandemic waves. More broadly, many available international studies focused on a single phase of the crisis, providing limited insight into whether psychological indicators and pregnancy-related COVID-19 stressors differed across pandemic waves within the same national context. This gap is particularly relevant in Central and Eastern Europe, including Hungary, where pandemic-related restrictions, healthcare access, and social circumstances may have shaped pregnant women’s experiences in specific ways.

Therefore, the present study aimed to examine depressive symptoms, hopelessness, self-efficacy, and pregnancy- and COVID-19-related stressors among pregnant women in Hungary during two distinct waves of the COVID-19 pandemic. The two-wave design was used to compare psychological and pandemic-related indicators across different stages of the pandemic, rather than to follow the same individuals over time. The study addressed three research questions: (1) whether depressive symptoms, hopelessness, self-efficacy differed between the two pandemic waves; (2) whether the identified pregnancy- and COVID-19-related stressor domains showed different patterns across waves; and (3) which demographic, pregnancy-related, psychological, and pandemic-related factors were associated with depressive symptoms and hopelessness within each wave.

## Methods

### Study design and participants

This study used a repeated cross-sectional design based on two independent online surveys conducted among pregnant women in Hungary during two distinct waves of the COVID-19 pandemic. The first data collection period took place between April and July 2020, corresponding to the first wave of the pandemic in Hungary, and the second between September 2020 and February 2021, corresponding to the second wave. Participants were not followed longitudinally; instead, the two survey waves were used to compare mental health indicators and pregnancy- and COVID-19-related stressors across different pandemic contexts. Eligibility criteria included being at least 18 years of age, being fluent in Hungarian, and being pregnant at the time of survey completion. Non-pregnant women were not recruited because the study focused on pregnancy-specific COVID-19 stressors, including concerns related to antenatal care, anticipated childbirth, and pregnancy-related healthcare experiences. The study focused on depressive symptoms, hopelessness, self-efficacy, and crisis-related concerns among pregnant women during the COVID-19 pandemic. Specifically, we compared psychological indicators and the identified stressor domains between the two waves, and examined which demographic, pregnancy-related, psychological, and pandemic-related factors were associated with depressive symptoms and hopelessness within each wave.

### Procedure

Before the main data collection, the survey was pretested among 128 pregnant women in the third trimester. These women were either hospitalized pregnant patients or pregnant women reached through the health-visitor network. The purpose of the pretest was not to conduct a separate psychometric validation study, but to assess the clarity, readability, comprehensibility, and perceived relevance of the questionnaire items. Participants were asked whether the items were understandable and easy to answer, and whether the content adequately reflected the difficulties and concerns they experienced during pregnancy in the COVID-19 pandemic context. The survey link was distributed through the Hungarian health-visitor network and through social media platforms and online communities related to pregnancy and motherhood. Health visitors were asked to forward the survey link to pregnant women under their care, and participants were also encouraged to share the link with other eligible pregnant women. Participation was voluntary and uncompensated. Before starting the questionnaire, participants received written information about the aims of the study, the voluntary nature of participation, anonymity and confidentiality, and their right to withdraw at any time before submitting their responses. Electronic informed consent was obtained before survey completion.

The study was approved by the Regional and Institutional Research Ethics Committee of the University of Szeged under approval number 69/2020-SZTE RKEB. All methods were performed in accordance with the relevant guidelines and regulations.

### Measures

The survey battery included sociodemographic and pregnancy-related questions, standardized psychological instruments assessing depressive symptoms, hopelessness, and self-efficacy, and the study-specific „Pregnancy during the coronavirus” questionnaire assessing pregnancy- and COVID-19-related concerns.

Sociodemographic and pregnancy-related data were collected at the beginning of the survey. Participants reported their current gestational week, age, place of residence, highest level of education, marital or relationship status, and duration of current relationship. Pregnancy-related questions assessed whether the current pregnancy was the participant’s first pregnancy, whether she already had children, the number of existing children, and whether the current pregnancy had been planned, unplanned, or unwanted.

General self-efficacy was assessed using the General Self-Efficacy Scale (GSE) developed by Schwarzer and Jerusalem^34^. The GSE is a 10-item, unidimensional self-report measure of perceived coping competence, reflecting the extent to which individuals believe they can manage challenging or stressful situations. Items are rated on a four-point Likert scale ranging from 1 („not at all true”) to 4 („exactly true”) (total score range: 10–40), with higher scores indicating greater perceived self-efficacy. The scale has shown good internal consistency in international samples, with Cronbach’s alpha values ranging from 0.76 to 0.90. The Hungarian version was adapted by Kopp and colleagues^35^. In the present study, the GSE was included to assess coping-related psychological resources in the context of pregnancy during the COVID-19 pandemic.

Hopelessness was assessed using the Beck Hopelessness Scale (BHS), originally developed by Beck and colleagues^36^ and adapted for use in Hungary by Perczel Forintos, Sallai, and Rózsa^37^. The BHS is a 20-item self-report questionnaire assessing negative expectations about the future, including feelings about the future, loss of motivation, and expectations. Items are answered in a true–false format with reference to the previous week, and are scored as 0 or 1, yielding a total score ranging from 0 to 20. Higher total scores indicate greater hopelessness. The scale includes seven reverse-scored items. According to the established scoring categories, scores of 4–8 indicate mild hopelessness, 9–14 moderate hopelessness, and 15–20 severe hopelessness^36^. The Hungarian adaptation showed good internal consistency, with a Cronbach’s alpha of 0.91^37^.

Depressive symptoms were assessed using the Edinburgh Postnatal Depression Scale (EPDS), originally developed by Cox, Holden, and Sagovsky^38^ as a 10-item self-report screening instrument for depressive symptoms in the postnatal period. Although initially developed for postnatal use, the EPDS has also been validated for use during pregnancy, including in a Hungarian antepartum sample^39^. The scale assesses depressive and anxiety-related symptoms experienced during the previous seven days. Items are rated on a four-point scale from 0 to 3, yielding a total score ranging from 0 to 30, with higher total scores indicating more severe depressive symptoms. The EPDS is a screening instrument and does not provide a clinical diagnosis^38^. The Hungarian antepartum validation showed acceptable internal consistency, with Cronbach’s alpha above 0.72. In the present study, EPDS scores were categorized according to cut-off values used for the antepartum period: scores of 0–7 indicated no clinically relevant depressive symptoms, scores of 8–12 indicated minor depressive symptoms, and scores of 13 or above indicated major depressive symptoms^39^. These categories were used for descriptive and comparative purposes only and were not interpreted as clinical diagnoses.

### Pregnancy during the coronavirus questionnaire

Pregnancy- and COVID-19-related concerns were assessed using the study-specific „Pregnancy during the coronavirus” questionnaire, which was developed for the present research. The questionnaire was designed to capture subjective concerns, stressors, and experiences related to being pregnant during the COVID-19 pandemic. Because limited directly relevant literature was available at the time of questionnaire development, item generation for Wave 1 was informed by the rapidly evolving Hungarian pandemic context, governmental public health measures and restrictions, changes in obstetric care, and professional experience from antenatal, obstetric, health-visitor, and psychological area^31,32,40^. The Wave 1 item pool was developed and reviewed by a multidisciplinary expert group. Obstetric expertise was provided by specialist obstetrician-gynaecologists and senior clinicians involved in the care of pregnant women, particularly with regard to antenatal care, childbirth, hospital procedures, and pregnancy-related healthcare risks. Health-visitor perspectives were provided by health visitors who had routine contact with pregnant women and were involved in distributing the questionnaire to the target population, as well as in bringing in practical experience from everyday pregnancy care. Psychological perspectives were provided by psychologists with relevant professional experience in maternal mental health, pregnancy-related distress, coping difficulties, and psychological burden. The role of the expert group was to ensure that the questionnaire covered topics that were clinically relevant and meaningful from the perspective of pregnant women’s everyday experiences during the early phase of the pandemic. These included uncertainty around antenatal care, anticipated childbirth, healthcare attendance, infection risk, fetal or newborn health, social support, family functioning, and everyday disruptions.

The item pool was further informed by concerns and experiences reported by pregnant women during the pretest. Similar or overlapping statements were merged, and redundant items were removed. The questionnaire was originally developed in Hungarian for use in the present Hungarian sample; therefore, no translation procedure was required. Items referred to the previous two weeks and covered several domains, including uncertainty related to antenatal care, anticipated childbirth-related concerns, concerns about the expected course and outcome of childbirth, the newborn’s health and possible infection, fear of infection and healthcare attendance, emotional and mood-related distress, perceived coping and social support, financial and housing concerns, social isolation and confinement, family and relationship tensions, media-related concerns, and difficulties in accessing services or professional support. Participants rated how true each statement felt for them on a four-point Likert scale ranging from 1 („not at all true”) to 4 („completely true”), with higher scores indicating stronger endorsement of the given concern or experience.

The Wave 1 version consisted of 41 items. For Wave 2, the questionnaire was reviewed and adapted by the same multidisciplinary expert group. The modification of the Wave 2 questionnaire was based on three main considerations. First, professional experience gained during Wave 1 data collection indicated that some concerns needed to be framed in relation to the earlier pandemic period. Second, Hungarian public health restrictions and obstetric care procedures had changed by the later phase of the pandemic, requiring some items to be reworded to reflect the current care context. Third, new concerns had become more relevant by Wave 2, including uncertainty compared with the first wave, optimism, adherence to protective measures, use of technological resources, trust in the healthcare system, and reliance on credible sources of information.

The same 41 core content areas were retained to preserve conceptual continuity across waves, while several items were reworded and six new items were added to capture these Wave 2-specific experiences. This resulted in a 47-item version used in Wave 2. The Wave 2 questionnaire should therefore be understood as an adapted version of the Wave 1 questionnaire rather than an identical repetition. Direct cross-wave comparisons were restricted to overlapping content areas or conceptually equivalent domains, and the six additional Wave 2 items were not treated as direct comparative indicators.

### Statistical analysis

Data analyses were conducted using IBM SPSS Statistics version 22.0. The online survey was configured so that responses to all mandatory items were required before submission. Therefore, there were no missing data for the variables included in the main analyses, and no imputation or missing-data replacement procedure was applied. Participants who did not meet the inclusion criteria, including those who reported being in the postpartum period rather than pregnant at the time of survey completion, were excluded from the final analytic sample.

The sample size was determined by the duration of the data-collection periods corresponding to two distinct COVID-19 pandemic waves in Hungary. These periods were aligned with the evolving epidemiological situation and the associated public health restrictions and measures, rather than by reaching a predetermined target sample size. Data collection was conducted within the relevant pandemic-wave periods and closed when the corresponding public health and social context had changed. The final analytic sample included 692 pregnant women in Wave 1 and 459 pregnant women in Wave 2, yielding a total analytic sample of 1,151 participants. These sample sizes were considered adequate for the exploratory PCA, group comparisons, correlation analyses, and multiple linear regression models conducted separately within each wave.

Descriptive statistics were used to characterize the sample and study variables. Continuous variables are reported as means and standard deviations, while categorical variables are reported as frequencies and percentages. EPDS categories were calculated from the EPDS total score using predefined antepartum cut-off values: 0–7 for no clinically relevant depressive symptoms, 8–12 for minor depressive symptoms, and ≥ 13 for major depressive symptoms. BHS categories were calculated according to established cut-off scores: 0–3 for none/minimal hopelessness, 4–8 for mild hopelessness, 9–14 for moderate hopelessness, and 15–20 for severe hopelessness. Chi-square tests were used to compare the distribution of EPDS and BHS categories across waves. Internal consistency of the standardized scales and the retained COVID-19-related questionnaire components was assessed using Cronbach’s alpha coefficient. The Beck Hopelessness Scale was scored after applying the standard reverse-scoring procedure for the relevant items.

For the study-specific COVID-19 questionnaire, principal component analysis (PCA) was used as an exploratory dimensionality-reduction method to identify interpretable COVID-19-related components within each wave. Principal component analyses with Varimax rotation were conducted separately for Wave 1 and Wave 2. The Wave 1 version of the questionnaire included 41 items, while the Wave 2 version included 47 items, including six additional items relevant to the later pandemic context. To retain a common item pool across waves, the six Wave 2-specific items were not included in the PCA. The item assessing difficulties in combining childcare, pregnancy, and work was conditional on having another child at home and was therefore excluded from the dimensionality analyses. Thus, PCA was conducted on the common, non-conditional COVID-19 questionnaire items in both waves. Kaiser–Meyer–Olkin values and Bartlett’s tests of sphericity were used to assess the suitability of the data for PCA. In Wave 1, two negatively worded help-seeking items were reverse-coded before calculating internal consistency and component scores for the perceived support and positive reappraisal component.

Because several psychological and COVID-19-related variables showed deviations from normality, non-parametric tests were used for group comparisons and correlation analyses. Potential demographic and pregnancy-related factors were examined through group comparisons and correlation analyses, including maternal age, gestational age, educational level, place of residence, relationship duration, first pregnancy status, having children, and pregnancy planning status. Mann–Whitney U tests were applied for two-group comparisons, including comparisons between Wave 1 and Wave 2 and comparisons according to binary demographic or pregnancy-related variables. Kruskal–Wallis tests were used for comparisons involving more than two groups, such as place of residence, educational level, and relationship duration. Where Kruskal–Wallis tests were significant and post hoc comparisons were appropriate, pairwise Mann–Whitney U tests with Bonferroni correction were conducted. Spearman’s rank correlations were used to examine associations between psychological measures, COVID-19-related component scores, maternal age, and gestational age.

Multiple linear regression analyses were conducted separately for Wave 1 and Wave 2 to identify predictors of depressive symptoms and hopelessness. EPDS and BHS total scores were modelled as separate outcomes. In each model, the other outcome variable, self-efficacy, the five COVID-19-related component scores, maternal age, and gestational age were entered simultaneously using the Enter method. Separate models were estimated for each wave because the data were collected during distinct pandemic periods and the COVID-19-related component structure was examined separately within each wave. Regression assumptions and diagnostics were examined before interpreting the models. Linearity and homoscedasticity were assessed by inspecting scatterplots of standardized residuals against standardized predicted values. The normality of residuals was evaluated using histograms and normal P–P plots of standardized residuals. Multicollinearity was assessed using tolerance and variance inflation factor values. Independence of residuals was examined using the Durbin-Watson statistic. Potentially influential cases were evaluated using standardized residuals and Cook’s distance. A significance level of p < 0.05 was adopted for all statistical tests, except for Bonferroni-corrected post hoc comparisons.

## Results

### Sample characteristics

A total of 694 respondents completed the online questionnaire in Wave 1, and 461 respondents completed it in Wave 2. During data screening, two respondents from each wave were excluded because they reported being in the postpartum period rather than pregnant at the time of completion. The final analytic sample therefore consisted of 692 pregnant women in Wave 1 and 459 pregnant women in Wave 2, resulting in a total sample of 1151 participants.

The sociodemographic and pregnancy-related characteristics of the sample are presented in Table 1. In Wave 1, the mean maternal age was 30.73 years (SD = 4.39), and the mean gestational age was 25.72 weeks (SD = 8.97; range: 5–41 weeks). In Wave 2, the mean maternal age was 31.19 years (SD = 4.38), and the mean gestational age was 24.88 weeks (SD = 9.41; range: 6–40 weeks). Across both waves, most participants lived in cities, had higher education qualifications, were married or living with a partner, and reported that their pregnancy was planned. In Wave 1, slightly more than half of the participants were in their first pregnancy (52.5%, n = 363), compared with 46.6% (n = 214) in Wave 2. The proportion of participants who already had at least one child was 42.8% (n = 296) in Wave 1 and 48.6% (n = 223) in Wave 2.

**Table 1:** Sociodemographic and pregnancy-related characteristics of participants by pandemic wave.

Characteristic		Wave 1 (n = 692)	Wave 2 (n = 459)
Maternal age, years, M (SD)		30.73 (4.39)	31.19 (4.38)
Gestational age, weeks, M (SD), range		25.72 (8.97), 5–41	24.88 (9.41), 6–40
Place of residence, n (%)	City	454 (65.6)	353 (76.9)
	Capital city	147 (21.2)	17 (3.7)
	Village	91 (13.2)	89 (19.4)
Educational level, n (%)	Less than secondary education	31 (4.5)	16 (3.5)
	Secondary education	208 (30.1)	165 (35.9)
	Higher education	453 (65.5)	278 (60.6)
Marital/relationship status, n (%)	Married	577 (83.4)	397 (86.5)
	Cohabiting/living with partner	113 (16.3)	61 (13.3)
	Single	2 (0.3)	-
	Divorced	-	1 (0.2)
Relationship duration, n (%)	Less than 1 year	10 (1.4)	2 (0.4)
	1–3 years	104 (15.0)	73 (15.9)
	4–6 years	225 (32.5)	124 (27.0)
	7–10 years	217 (31.4)	135 (29.4)
	11–15 years	105 (15.2)	101 (22.0)
	More than 15 years	29 (4.2)	23 (5.0)
	No current relationship	2 (0.3)	1 (0.2)
First pregnancy, n (%)	No	329 (47.5)	245 (53.4)
	Yes	363 (52.5)	214 (46.6)
Already has children, n (%)	No	396 (57.2)	236 (51.4)
	Yes	296 (42.8)	223 (48.6)
Pregnancy planning, n (%)	Planned	645 (93.2)	424 (92.4)
	Unplanned	46 (6.6)	34 (7.4)
	Unwanted	1 (0.1)	1 (0.2)

Values are presented as n (%) unless otherwise indicated. M = mean; SD = standard deviation. Percentages are calculated within each wave and may not sum to 100 because of rounding. A dash indicates that no participant was present in that category.

### Psychological characteristics and wave comparisons

Descriptive statistics for the psychological measures indicated broadly similar patterns across the two waves. The mean EPDS score was 6.50 (SD = 4.83; range: 0–25) in Wave 1 and 6.06 (SD = 4.81; range: 0–30) in Wave 2. Mean self-efficacy scores were also comparable across waves, with 32.76 (SD = 5.06; range: 14–40) in Wave 1 and 32.89 (SD = 5.17; range: 13–40) in Wave 2. After applying the standard reverse-scoring procedure for the Beck Hopelessness Scale, the mean BHS score was 3.51 (SD = 2.00; range: 0–16) in Wave 1 and 3.39 (SD = 2.11; range: 1–17) in Wave 2. Because psychological scale scores deviated from normality, Mann–Whitney U tests were used to compare continuous psychological measures between the two independent waves. EPDS scores did not differ significantly between Wave 1 and Wave 2 (U = 150,043.00, Z = −1.59, p = 0.111). Self-efficacy scores also did not differ significantly between waves (U = 155,730.00, Z = −0.56, p = 0.576). The difference in BHS scores was at the conventional significance threshold (U = 148,442.00, Z = −1.96, p = 0.050). Chi-square tests were used to compare EPDS and BHS categories across waves. The distribution of EPDS categories did not differ significantly between Wave 1 and Wave 2 (χ^2^(2, N = 1151) = 5.257, p = 0.072). In Wave 1, 63.3% of participants were classified as having no clinically relevant depressive symptoms, 24.9% fell into the minor depressive symptom range, and 11.8% into the major depressive symptom range. In Wave 2, the corresponding proportions were 69.7%, 19.8%, and 10.5%, respectively. For BHS categories, moderate and severe hopelessness were combined because of low cell counts. The distribution of BHS categories did not differ significantly between waves (χ^2^(2, N = 1151) = 2.973, p = 0.226). In Wave 1, 65.8% of participants showed none/minimal hopelessness, 31.1% mild hopelessness, and 3.2% moderate/severe hopelessness. In Wave 2, the corresponding proportions were 70.4%, 26.4%, and 3.3%, respectively.

### Principal component analysis of the COVID-19 questionnaire

Principal component analysis (PCA) was conducted separately for Wave 1 and Wave 2 using the common, non-conditional COVID-19 questionnaire items. The aim was to identify interpretable COVID-19-related stressor domains within each pandemic wave. The item assessing difficulties in combining childcare, pregnancy, and work was excluded from the dimensionality analyses because it was conditional on having another child and was therefore not applicable to all participants. The six additional items introduced only in Wave 2 were also excluded to retain the same common item pool across waves. Thus, both PCA analyses were conducted on 40 items.

The data were suitable for PCA in both waves. In Wave 1, the Kaiser–Meyer–Olkin value was 0.925, and Bartlett’s test of sphericity was significant (χ^2^(780) = 13,508.923, p < 0.001). In Wave 2, the corresponding indices were similarly adequate, KMO = 0.930, Bartlett’s χ^2^(780) = 9726.562, p < 0.001. A five-component solution with Varimax rotation was retained in both waves. This solution explained 51.61% of the total variance in Wave 1 and 53.39% in Wave 2. After rotation, the five components accounted for 16.70%, 14.52%, 7.60%, 6.61%, and 6.17% of the variance in Wave 1, and 20.27%, 12.50%, 7.28%, 6.83%, and 6.51% in Wave 2, respectively. Across both waves, the retained components reflected emotional distress and impaired coping; pregnancy-, childbirth-, and healthcare-related concerns; confinement-related difficulties and daily disruption; perceived support and positive reappraisal; and financial and housing concerns. The component labels, main item content, variance explained after rotation, and internal consistency values are presented in Table 2. In Wave 1, two negatively worded help-seeking items were reverse-coded before calculating internal consistency and component scores for the perceived support and positive reappraisal component. In Wave 2, these items did not load on the perceived support and positive reappraisal component; therefore, no reverse coding was applied to these items when calculating the Wave 2 component score.

**Table 2:** Retained principal components of the common COVID-19 questionnaire items by pandemic wave.

Wave	Component	Component label	Main item content	Variance explained after rotation	Cronbach’s α
Wave 1	1	Emotional distress and impaired coping	Low mood, loss of pleasure, tension, concentration difficulties, reduced perceived usefulness, sleep difficulties, domestic tension, feelings of being left alone	16.70%	0.919
Wave 1	2	Pregnancy-, childbirth-, and healthcare-related concerns	Childbirth uncertainty, fear of giving birth alone, fear of attending healthcare settings, COVID-related health concerns, difficulty accessing professional support, concerns about family members’ health, reluctance to ask for help	14.52%	0.875
Wave 1	3	Confinement-related difficulties and daily disruption	Difficulties tolerating confinement and restrictions, lack of daily structure, difficulty accessing basic services or goods	7.60%	0.788
Wave 1	4	Perceived support and positive reappraisal	Perceived availability of support, knowing whom to turn to, reduced fear of rejection when asking for help, positive reappraisal of the pandemic situation	6.61%	0.653
Wave 1	5	Financial and housing concerns	Fear of job loss, fear of financial difficulties, actual financial problems, housing difficulties	6.17%	0.705
Wave 2	1	Emotional distress and impaired coping	Low mood, loss of pleasure, tension, concentration difficulties, self-blame, perceived inability to cope, sleep difficulties, perceived uselessness, lack of daily orientation, fear of being unable to care for the child alone, reluctance to ask for help, fear of rejection when seeking support	20.27%	0.931
Wave 2	2	Pregnancy-, childbirth-, and healthcare-related concerns	Childbirth uncertainty, inability to prepare a birth plan, lack of family support after birth, fear of giving birth alone, fear of attending healthcare settings, COVID symptom monitoring, uncertainty about whom to contact, perceived abandonment, difficulty accessing services or goods, concern caused by media coverage	12.50%	0.864
Wave 2	3	Confinement-related difficulties and daily disruption	Difficulties tolerating confinement, increased relationship or domestic tensions, lack of daily structure, difficulties tolerating restrictions	7.28%	0.730
Wave 2	4	Financial and housing concerns	Fear of financial difficulties, actual financial problems, fear of job loss, housing difficulties	6.83%	0.749
Wave 2	5	Perceived support and positive reappraisal	Positive reappraisal of the pandemic situation, perceived availability of support, opportunities for self-focus, knowing whom to turn to, concern for close others	6.51%	0.659

PCA = principal component analysis. Components were derived from the common, non-conditional COVID-19 questionnaire items. In this pregnant sample, childbirth-related concerns refer to anticipated concerns about childbirth, including worries about giving birth, the expected course of childbirth, and the newborn’s health. Cronbach’s α values indicate the internal consistency of the retained item sets.

### COVID-19-related component scores

Descriptive statistics and Mann–Whitney U test results for the COVID-19-related component scores are presented in Table 3. Significant differences between waves were found for confinement-related difficulties and daily disruption (U = 117,745.00, Z = −7.45, p < 0.001), and perceived support and positive reappraisal (U = 111,145.50, Z = −8.66, p < 0.001). Mean scores for both components were higher in Wave 1 than in Wave 2. No significant differences were found for emotional distress and impaired coping, pregnancy-, childbirth-, and healthcare-related concerns, or financial and housing concerns.

**Table 3:** COVID-19-related component scores and wave comparisons.

Component	Wave 1 M (SD)	Wave 1 range	Wave 2 M (SD)	Wave 2 range	Mann–Whitney U	Z	p
Emotional distress and impaired coping	1.52 (0.58)	1.00–3.69	1.58 (0.62)	1.00–3.93	152,205.50	−1.20	0.230
Pregnancy-, childbirth-, and healthcare-related concerns	2.13 (0.69)	1.00–3.92	2.17 (0.69)	1.00–4.00	152,858.00	−1.08	0.281
Confinement-related difficulties and daily disruption	2.31 (0.82)	1.00–4.00	1.94 (0.67)	1.00–4.00	117,745.00	−7.45	< 0.001
Perceived support and positive reappraisal	3.39 (0.52)	1.33–4.00	3.09 (0.62)	1.00–4.00	111,145.50	−8.66	< 0.001
Financial and housing concerns	1.51 (0.58)	1.00–4.00	1.60 (0.65)	1.00–4.00	148,665.00	−1.88	0.060

Component scores were calculated as mean scores of the retained items within each component. Higher scores indicate higher levels of the respective component. for perceived support and positive reappraisal, higher scores indicate greater perceived support and more positive reappraisal. Childbirth-related concerns refer to anticipated concerns during pregnancy. Mann–Whitney U tests were used to compare Wave 1 and Wave 2 scores. M = mean; SD = standard deviation; U = Mann–Whitney test statistic; Z = standardized test statistic; p = two-tailed significance value.

### Correlations between psychological measures and COVID-19-related component scores

Spearman’s rank correlation analyses were conducted separately for Wave 1 and Wave 2 to examine associations between psychological measures, COVID-19-related component scores, maternal age, and gestational age. The main correlation coefficients are presented in Table 4. In both waves, EPDS scores showed the strongest positive associations with emotional distress and impaired coping (Wave 1: ρ = 0.711, p < 0.001; Wave 2: ρ = 0.641, p < 0.001). EPDS scores were also positively associated with pregnancy-, childbirth-, and healthcare-related concerns, confinement-related difficulties and daily disruption, and financial and housing concerns in both waves. A negative association between EPDS scores and perceived support and positive reappraisal was observed in Wave 1 (ρ = −0.361, p < 0.001), but not in Wave 2 (ρ = −0.042, p = 0.367).

**Table 4:** Spearman correlations of psychological measures with COVID-19-related component scores and age variables by pandemic wave.

Wave	Variable	ED/IC	PCHC	CDD	PSPR	FHC	Maternal age	Gestational age
Wave 1	EPDS	0.711***	0.561***	0.485***	−0.361***	0.275***	−0.056	0.064
Wave 1	BHS	0.301***	0.164***	0.152***	−0.256***	0.180***	0.015	0.008
Wave 1	GSE	−0.375***	−0.285***	−0.189***	0.282***	−0.179***	0.058	−0.039
Wave 2	EPDS	0.641***	0.484***	0.494***	−0.042	0.395***	0.008	−0.018
Wave 2	BHS	0.213***	0.157**	0.158**	−0.169***	0.223***	0.107*	0.012
Wave 2	GSE	−0.330***	−0.216***	−0.220***	0.190***	−0.254***	−0.008	−0.015

Values are Spearman’s rho coefficients (ρ). EPDS = Edinburgh Postnatal Depression Scale; BHS = Beck Hopelessness Scale; GSE = General Self-Efficacy Scale. ED/IC = emotional distress and impaired coping; PCHC = pregnancy-, childbirth-, and healthcare-related concerns; CDD = confinement-related difficulties and daily disruption; PSPR = perceived support and positive reappraisal; FHC = financial and housing concerns. Childbirth-related concerns refer to anticipated concerns during pregnancy. *p < 0.05, **p < 0.01, ***p < 0.001.

BHS scores showed weaker but significant positive associations with emotional distress and impaired coping in both waves (Wave 1: ρ = 0.301, p < 0.001; Wave 2: ρ = 0.213, p < 0.001), as well as with pregnancy-, childbirth-, and healthcare-related concerns, confinement-related difficulties and daily disruption, and financial and housing concerns. BHS scores were negatively associated with perceived support and positive reappraisal in both waves (Wave 1: ρ = −0.256, p < 0.001; Wave 2: ρ = −0.169, p < 0.001). Self-efficacy showed the opposite pattern, with negative associations with emotional distress and impaired coping (Wave 1: ρ = −0.375, p < 0.001; Wave 2: ρ = −0.330, p < 0.001) and positive associations with perceived support and positive reappraisal in both waves (Wave 1: ρ = 0.282, p < 0.001; Wave 2: ρ = 0.190, p < 0.001).

Maternal age and gestational age showed no consistent associations with the main psychological outcomes. Maternal age was weakly positively associated only with BHS scores in Wave 2 (ρ = 0.107, p < 0.05). Gestational age was not significantly associated with EPDS, BHS, or self-efficacy scores in either wave. Regarding COVID-19-related components, gestational age showed weak associations with pregnancy-, childbirth-, and healthcare-related concerns in Wave 1 (ρ = 0.129, p = 0.001), perceived support and positive reappraisal in Wave 1 (ρ = −0.075, p = 0.048), and perceived support and positive reappraisal in Wave 2 (ρ = −0.093, p = 0.047).

### Sociodemographic and pregnancy-related group differences

Mann–Whitney U tests and Kruskal–Wallis tests were conducted separately for Wave 1 and Wave 2 to examine whether psychological measures and COVID-19-related component scores differed according to pregnancy-related and sociodemographic characteristics.

#### First pregnancy status

In Wave 1, BHS scores differed significantly by first pregnancy status (U = 49,031.00, Z = −4.23, p < 0.001), with higher mean ranks among participants who were not in their first pregnancy. First pregnancy status was also associated with pregnancy-, childbirth-, and healthcare-related concerns (U = 54,515.00, Z = −1.98, p = 0.048), confinement-related difficulties and daily disruption (U = 51,844.50, Z = −3.01, p = 0.003), and financial and housing concerns (U = 52,408.00, Z = −2.85, p = 0.004). These COVID-19-related component scores showed higher mean ranks among participants in their first pregnancy. No significant differences were found for EPDS scores, self-efficacy, emotional distress and impaired coping, or perceived support and positive reappraisal in Wave 1. In Wave 2, first pregnancy status was significantly associated only with BHS scores (U = 23,079.00, Z = −2.31, p = 0.021), again with higher mean ranks among participants who were not in their first pregnancy. No significant differences by first pregnancy status were found for EPDS scores, self-efficacy, or any of the COVID-19-related component scores in Wave 2.

#### Having children

In Wave 1, BHS scores differed significantly according to whether participants already had children (U = 48,680.00, Z = −3.97, p < 0.001), with higher mean ranks among participants who already had children. Significant group differences were also found for confinement-related difficulties and daily disruption (U = 51,552.00, Z = −2.72, p = 0.006), perceived support and positive reappraisal (U = 52,625.50, Z = −2.32, p = 0.021), and financial and housing concerns (U = 52,629.50, Z = −2.36, p = 0.018). For these COVID-19-related components, higher mean ranks were observed among participants who did not yet have children. No significant differences were found for EPDS scores, self-efficacy, emotional distress and impaired coping, or pregnancy-, childbirth-, and healthcare-related concerns in Wave 1. In Wave 2, no significant differences were found according to whether participants already had children for EPDS scores, BHS scores, self-efficacy, or any of the COVID-19-related component scores.

#### Pregnancy planning status

Because the unwanted pregnancy category included very few participants, unplanned and unwanted pregnancies were combined and compared with planned pregnancies. In Wave 1, no significant differences were found between planned and unplanned/unwanted pregnancies for EPDS scores, BHS scores, self-efficacy, or any of the COVID-19-related component scores. In Wave 2, significant group differences were found for EPDS scores (U = 5832.00, Z = −2.11, p = 0.035), BHS scores (U = 5459.50, Z = −2.72, p = 0.007), and financial and housing concerns (U = 5787.00, Z = −2.21, p = 0.027). For these variables, higher mean ranks were observed among participants with unplanned or unwanted pregnancies. No significant differences were found for self-efficacy, emotional distress and impaired coping, pregnancy-, childbirth-, and healthcare-related concerns, confinement-related difficulties and daily disruption, or perceived support and positive reappraisal in Wave 2.

#### Place of residence

Kruskal–Wallis tests showed that place of residence was significantly associated only with self-efficacy in both waves. In Wave 1, self-efficacy differed by place of residence (χ^2^(2) = 6.398, p = 0.041), with the lowest mean ranks among participants living in the capital city and the highest among those living in villages. No significant differences by place of residence were found for EPDS scores, BHS scores, or any of the COVID-19-related component scores in Wave 1. In Wave 2, place of residence was again significantly associated only with self-efficacy (χ^2^(2) = 10.962, p = 0.004), with the highest mean ranks among participants living in the capital city and the lowest among those living in villages. No significant differences were found for EPDS scores, BHS scores, or any of the COVID-19-related component scores in Wave 2. Given the small number of participants living in the capital city in Wave 2 (n = 17).

#### Educational level

Educational level was categorized as less than secondary education, secondary education, and higher education. For significant Kruskal–Wallis tests, post hoc pairwise Mann–Whitney U tests with Bonferroni correction were conducted. In Wave 1, significant differences by educational level were found for BHS scores (χ^2^(2) = 8.872, p = 0.012), self-efficacy (χ^2^(2) = 6.324, p = 0.042), confinement-related difficulties and daily disruption (χ^2^(2) = 12.816, p = 0.002), and financial and housing concerns (χ^2^(2) = 11.423, p = 0.003). Post hoc comparisons showed that participants with less than secondary education had higher BHS scores (U = 5120.50, Z = −2.62, p = 0.009), and higher self-efficacy scores (U = 5166.00, Z = −2.47, p = 0.014), than participants with higher education. Participants with secondary education had higher scores on confinement-related difficulties and daily disruption (U = 39,229.50, Z = −3.47, p = 0.001), and financial and housing concerns (U = 40,479.00, Z = −2.99, p = 0.003), than participants with higher education. No other pairwise comparisons remained significant after Bonferroni correction. In Wave 2, significant differences by educational level were found for emotional distress and impaired coping (χ^2^(2) = 8.673, p = 0.013), and perceived support and positive reappraisal (χ^2^(2) = 8.025, p = 0.018). Pregnancy-, childbirth-, and healthcare-related concerns were at the conventional significance threshold (χ^2^(2) = 5.985, p = 0.050). Post hoc comparisons showed that perceived support and positive reappraisal was higher among participants with secondary education than among those with less than secondary education (U = 796.00, Z = −2.63, p = 0.008), and also higher among participants with higher education than among those with less than secondary education (U = 1296.00, Z = −2.82, p = 0.005). Emotional distress and impaired coping was higher among participants with higher education than among those with secondary education (U = 19,334.00, Z = −2.77, p = 0.006). The difference in pregnancy-, childbirth-, and healthcare-related concerns between participants with secondary and higher education was at the Bonferroni-corrected significance threshold (U = 19,840.50, Z = −2.38, p = 0.017), with higher mean ranks among participants with higher education. No other pairwise comparisons remained significant after Bonferroni correction.

#### Relationship duration

In Wave 1, relationship duration was significantly associated only with self-efficacy (χ^2^(6) = 13.611, p = 0.034). Because relationship-duration categories were unevenly distributed and the non-partnered group included only two participants, post hoc pairwise comparisons were not interpreted. No significant differences by relationship duration were found for EPDS scores, BHS scores, or any of the COVID-19-related component scores in Wave 1. In Wave 2, relationship duration was not significantly associated with EPDS scores, BHS scores, self-efficacy, or any of the COVID-19-related component scores.

### Multiple linear regression models predicting EPDS scores

Multiple linear regression analyses were conducted separately for Wave 1 and Wave 2 to identify predictors of EPDS scores. In both models, BHS scores, self-efficacy, the five COVID-19-related component scores, maternal age, and gestational age were entered simultaneously using the Enter method. Table 5 summarizes the significant predictors identified in the Wave 1 and Wave 2 EPDS models.

**Table 5:** Significant predictors of EPDS scores in the Wave 1 and Wave 2 multiple linear regression models.

Wave	Predictor	B	SE B	β	t	p	95% CI for B
Wave 1	GSE total score	−0.112	0.026	−0.117	−4.305	< 0.001	−0.162 to −0.061
Wave 1	Emotional distress and impaired coping	4.676	0.327	0.562	14.313	< 0.001	4.034 to 5.317
Wave 1	Pregnancy-, childbirth-, and healthcare-related concerns	0.982	0.231	0.140	4.251	< 0.001	0.529 to 1.436
Wave 1	Confinement-related difficulties and daily disruption	0.414	0.189	0.071	2.192	0.029	0.043 to 0.784
Wave 2	BHS total score	0.470	0.089	0.206	5.294	< 0.001	0.295 to 0.644
Wave 2	GSE total score	−0.093	0.034	−0.100	−2.709	0.007	−0.160 to −0.025
Wave 2	Emotional distress and impaired coping	3.236	0.437	0.417	7.410	< 0.001	2.378 to 4.094
Wave 2	Confinement-related difficulties and daily disruption	0.856	0.321	0.120	2.667	0.008	0.225 to 1.487
Wave 2	Financial and housing concerns	0.565	0.278	0.077	2.027	0.043	0.017 to 1.112

Only statistically significant predictors are displayed. EPDS = Edinburgh Postnatal Depression Scale; BHS = Beck Hopelessness Scale; GSE = General Self-Efficacy Scale; B = unstandardized regression coefficient; SE B = standard error of B; β = standardized regression coefficient; t = t-test statistic for the regression coefficient; p = significance value; CI = confidence interval. Childbirth-related concerns refer to anticipated concerns during pregnancy. All predictors were entered simultaneously using the Enter method. The full models included BHS total scores, GSE total scores, five COVID-19-related component scores, maternal age, and gestational age.

In Wave 1, the regression model was significant (F(9, 682) = 107.084, p < 0.001), explaining 58.6% of the variance in EPDS scores (R^2^ = 0.586, adjusted R^2^ = 0.580). Higher emotional distress and impaired coping (β = 0.562, p < 0.001), pregnancy-, childbirth-, and healthcare-related concerns (β = 0.140, p < 0.001), and confinement-related difficulties and daily disruption (β = 0.071, p = 0.029), were significant positive predictors of EPDS scores. Higher self-efficacy was a significant negative predictor (β = −0.117, p < 0.001). BHS scores, perceived support and positive reappraisal, financial and housing concerns, maternal age, and gestational age were not significant predictors in the Wave 1 model. In Wave 2, the regression model was also significant (F(9, 449) = 50.819, p < 0.001), explaining 50.5% of the variance in EPDS scores (R^2^ = 0.505, adjusted R^2^ = 0.495). Higher BHS scores (β = 0.206, p < 0.001), emotional distress and impaired coping (β = 0.417, p < 0.001), confinement-related difficulties and daily disruption (β = 0.120, p = 0.008), and financial and housing concerns (β = 0.077, p = 0.043), were significant positive predictors. Higher self-efficacy was again a significant negative predictor (β = −0.100, p = 0.007). Pregnancy-, childbirth-, and healthcare-related concerns, perceived support and positive reappraisal, maternal age, and gestational age were not significant predictors in the Wave 2 model.

### Multiple linear regression models predicting BHS scores

The same modelling strategy was applied to BHS scores as the outcome variable. In these models, EPDS scores, self-efficacy, the five COVID-19-related component scores, maternal age, and gestational age were entered simultaneously. Significant predictors for hopelessness in Wave 1 and Wave 2 are shown in Table 6.

**Table 6:** Significant predictors of BHS scores in the Wave 1 and Wave 2 multiple linear regression models.

Wave	Predictor	B	SE B	β	t	p	95% CI for B
Wave 1	GSE total score	−0.058	0.014	−0.146	−4.097	< 0.001	−0.085 to −0.030
Wave 1	Emotional distress and impaired coping	1.588	0.193	0.460	8.230	< 0.001	1.209 to 1.967
Wave 1	Confinement-related difficulties and daily disruption	−0.236	0.103	−0.097	−2.302	0.022	−0.437 to −0.035
Wave 1	Perceived support and positive reappraisal	−0.378	0.148	−0.098	−2.562	0.011	−0.669 to −0.088
Wave 2	EPDS total score	0.125	0.024	0.285	5.294	< 0.001	0.079 to 0.171
Wave 2	GSE total score	−0.043	0.018	−0.107	−2.455	0.014	−0.078 to −0.009
Wave 2	Emotional distress and impaired coping	1.108	0.233	0.325	4.757	< 0.001	0.650 to 1.565
Wave 2	Perceived support and positive reappraisal	−0.486	0.137	−0.142	−3.546	< 0.001	−0.756 to −0.217

Only statistically significant predictors are displayed. BHS = Beck Hopelessness Scale; EPDS = Edinburgh Postnatal Depression Scale; GSE = General Self-Efficacy Scale; B = unstandardized regression coefficient; SE B = standard error of B; β = standardized regression coefficient; t = t-test statistic for the regression coefficient; p = significance value; CI = confidence interval. All predictors were entered simultaneously using the Enter method. The full models included EPDS total scores, GSE total scores, five COVID-19-related component scores, maternal age, and gestational age.

In Wave 1, the regression model was significant (F(9, 682) = 30.909, p < 0.001), explaining 29.0% of the variance in BHS scores (R^2^ = 0.290, adjusted R^2^ = 0.280). Higher emotional distress and impaired coping was the strongest positive predictor of BHS scores (β = 0.460, p < 0.001). Higher self-efficacy, (β = −0.146, p < 0.001), confinement-related difficulties and daily disruption (β = −0.097, p = 0.022), and perceived support and positive reappraisal (β = −0.098, p = 0.011), were significant negative predictors. EPDS scores, pregnancy-, childbirth-, and healthcare-related concerns, financial and housing concerns, maternal age, and gestational age were not significant predictors in the Wave 1 model.

In Wave 2, the regression model was also significant (F(9, 449) = 23.082, p < 0.001), explaining 31.6% of the variance in BHS scores (R^2^ = 0.316, adjusted R^2^ = 0.303). Higher EPDS scores (β = 0.285, p < 0.001), and higher emotional distress and impaired coping (β = 0.325, p < 0.001), were significant positive predictors of BHS scores. Higher self-efficacy (β = −0.107, p = 0.014), and perceived support and positive reappraisal (β = −0.142, p < 0.001), were significant negative predictors. Pregnancy-, childbirth-, and healthcare-related concerns, confinement-related difficulties and daily disruption, financial and housing concerns, maternal age, and gestational age were not significant predictors in the Wave 2 model.

Regression diagnostics were examined for all EPDS and BHS regression models. No problematic multicollinearity was detected. In the EPDS models, VIF values ranged from 1.033 to 2.538 in Wave 1 and from 1.017 to 2.871 in Wave 2. In the BHS models, VIF values ranged from 1.031 to 3.002 in Wave 1 and from 1.014 to 3.068 in Wave 2. Durbin-Watson values were close to 2 across all models, ranging from 1.835 to 2.121. Inspection of residual plots did not indicate severe violations of homoscedasticity. Some deviations from residual normality and several standardized residuals exceeding ± 3 were observed, particularly in Wave 2, which is consistent with the bounded and skewed distribution of the psychological scale scores. However, Cook’s distance values remained below 1 in all models. Maximum Cook’s distance values were 0.049 and 0.582 in the Wave 1 and Wave 2 EPDS models, respectively, and 0.091 and 0.295 in the Wave 1 and Wave 2 BHS models, respectively. Therefore, no highly influential cases were identified.

## Discussion

The present study examined depressive symptoms, hopelessness, self-efficacy, and pregnancy- and COVID-19-related stressor domains among pregnant women in Hungary during two distinct waves of the COVID-19 pandemic. By using two independent cross-sectional samples, the study provides insight into how psychological indicators and pandemic-related concerns appeared across two different pandemic contexts, rather than describing within-person change over time.

Overall, depressive symptom categories did not differ significantly between the two waves. In both samples, approximately one in ten participants fell into the major depressive symptom range, while about one fifth to one quarter reported minor depressive symptoms. These findings are consistent with international evidence showing that depressive symptoms were common among pregnant women during the COVID-19 pandemic, although prevalence estimates varied across countries, measurement approaches, and pandemic contexts^22–25^. Compared with studies reporting markedly elevated distress in some pregnant samples^24^^.^^25^, the present findings suggest a more moderate, but still clinically meaningful, depressive symptom burden. In European samples, Fransson et al. reported positive depressive symptom screening rates of 18.3% during the first and 20.1% during the second Swedish pandemic wave^29^, whereas Favre et al. found substantially higher rates in Switzerland, with 32.9% of pregnant individuals reporting severe depressive symptoms and 37.0% meeting criteria for mental health impairment^30^. The Hungarian findings therefore appear to fall between lower-to-moderate European estimates and the more severe distress levels reported in some online samples. The absence of a significant difference between waves may indicate that depressive symptom burden remained present across different phases of the pandemic, although the stress context changed: the first wave was characterized by abrupt uncertainty and disruption, whereas the second involved prolonged pandemic exposure and higher epidemiological burden.

Hopelessness showed a similar pattern. The distribution of BHS categories did not differ significantly between waves, and moderate/severe hopelessness was rare in both samples. Nevertheless, mild hopelessness was reported by a substantial minority of participants. Although hopelessness has been less frequently examined than depression or anxiety in COVID-19 studies among pregnant women, it is conceptually relevant because it reflects negative expectations about the future, reduced perceived control, and potentially lower help-seeking motivation. This aligns with previous findings showing that pregnancy- and fetal-health-related COVID-19 worries were associated with poorer mental health among pregnant individuals^30^. In pregnancy, such future-oriented negative expectations may be especially important, as women were required to make decisions about healthcare, childbirth preparation, and family support under uncertain pandemic conditions.

The PCA of the study-specific COVID-19 questionnaire identified five interpretable components in both waves: emotional distress and impaired coping; pregnancy-, childbirth-, and healthcare-related concerns; confinement-related difficulties and daily disruption; perceived support and positive reappraisal; and financial and housing concerns. These components reflect domains repeatedly highlighted in previous pandemic studies, including fear of infection, disruption of antenatal care, uncertainty about childbirth, social isolation, and practical or financial difficulties^7,22,30^. The component structure is also consistent with early pandemic studies that used study-specific COVID-19 concern items to capture rapidly emerging stressors among pregnant women^30^. The identification of conceptually similar components across waves suggests that these domains represented recurring dimensions of pregnant women’s pandemic-related experiences, although they should be interpreted as exploratory stressor domains rather than as a previously established measurement model.

Among the COVID-19-related components, confinement-related difficulties and daily disruption were significantly higher in Wave 1 than in Wave 2. This difference can be understood in relation to the Hungarian public health context, as the first wave involved the rapid introduction of restrictions affecting healthcare visits, mobility, social contact, childcare and education, public events, services, and daily routines^31,32^. For pregnant women, these abrupt changes may have disrupted access to support, childcare arrangements, and expectations regarding antenatal care and childbirth. By contrast, the second wave was characterized by a substantially higher epidemiological burden and renewed restrictions, but by this stage the pandemic context and many protective measures may have become more familiar. This may partly explain why confinement-related difficulties and daily disruption were reported as higher in Wave 1 despite the more severe epidemiological context of Wave 2. Perceived support and positive reappraisal were also higher in Wave 1, which may reflect stronger initial mobilization of family and social support, the perception of the first lockdown as a shared and temporary crisis, or more active attempts to reframe the situation^31,32^. Because the two waves included independent samples, these differences should not be interpreted as evidence of within-person adaptation over time, but rather as reflecting two distinct epidemiological, social, and healthcare contexts in Hungary.

The correlation analyses showed that depressive symptoms were most strongly associated with emotional distress and impaired coping in both waves. This component included affective and cognitive experiences closely related to depressive symptomatology, such as low mood, loss of pleasure, tension, sleep difficulties, concentration problems, and perceived inability to cope. EPDS scores were also positively associated with pregnancy-, childbirth-, and healthcare-related concerns, confinement-related difficulties, and financial and housing concerns. These associations suggest that depressive symptoms during pregnancy were embedded in a broader pandemic-related stress context, in which worries about antenatal care, anticipated childbirth, fetal or newborn health, social restrictions, disrupted routines, and material insecurity may have co-occurred with poorer emotional well-being. This is consistent with prior evidence indicating that pandemic-related uncertainty, fear of infection, and disruptions in prenatal care were associated with poorer mental health among pregnant women^7,22,30^.

Hopelessness showed a similar, although weaker, pattern of associations. Higher BHS scores were related to emotional distress and impaired coping, pregnancy-, childbirth-, and healthcare-related concerns, confinement-related difficulties, and financial and housing concerns in both waves. These findings may reflect the future-oriented nature of hopelessness: uncertainty about healthcare access, childbirth conditions, family support, infection risk, and financial stability may have been experienced not only as current stressors, but also as threats to future safety and predictability. Conversely, women who perceived more available support or were more able to reframe the situation positively reported fewer hopeless expectations. Self-efficacy showed the opposite pattern: it was negatively associated with distress- and stressor-related components and positively associated with perceived support and positive reappraisal. This is consistent with the role of self-efficacy as a psychological resource that may support perceived coping in stressful circumstances^34,35^. However, given the cross-sectional design, these associations should be interpreted as concurrent relationships rather than evidence that self-efficacy protected against distress.

The group comparison analyses further indicated that some pregnancy-related and sociodemographic characteristics were associated with psychological or pandemic-related indicators. In Wave 1, participants who were not in their first pregnancy and those who already had children reported higher hopelessness, whereas several COVID-19-related concerns were higher among women in their first pregnancy or without children. This pattern may reflect different forms of vulnerability during the early pandemic period. Women who already had children may have faced additional caregiving and household responsibilities at a time when childcare institutions and schools were closed or shifted to digital education, daily routines were disrupted, and family support was constrained by distancing measures^32,32^. By contrast, first-time pregnant women or those without children may have experienced greater uncertainty regarding antenatal care, anticipated childbirth, hospital restrictions, and the health of the fetus or newborn, which is consistent with previous findings identifying pregnancy-specific worries, fear of infection, and disruption of prenatal care as key stressors during the pandemic^7,22,24,31^.

In Wave 2, unplanned or unwanted pregnancy was associated with higher depressive symptoms, hopelessness, and financial and housing concerns. This finding may be particularly relevant in the context of the second wave, when the epidemiological burden was substantially higher and renewed restrictions affected work, services, hospitality, education, and social contact^32^. For women whose pregnancy was unplanned or unwanted, pandemic-related economic insecurity, reduced access to informal support, and uncertainty about future family resources may have been experienced as especially burdensome. These findings suggest that pregnancy planning status, existing caregiving responsibilities, and material insecurity should be considered when assessing psychological vulnerability among pregnant women during public health crises.

Educational differences were also observed, although their pattern varied by wave and outcome. In Wave 1, lower educational level was associated with higher BHS scores and higher self-efficacy, while secondary education was associated with higher confinement-related and financial/housing concerns compared with higher education. In Wave 2, emotional distress and impaired coping were higher among participants with higher education than among those with secondary education, while perceived support and positive reappraisal were lower among those with less than secondary education. These mixed findings suggest that educational level did not function as a simple linear risk or protective factor. Rather, it may have captured broader contextual differences in occupational situation, financial vulnerability, information exposure, health literacy, access to support, and work–family demands during the pandemic. Similar complexity has been reported in previous pandemic studies, where socioeconomic conditions, social support, isolation, and healthcare access were linked to maternal mental health during pregnancy^22–25^. Therefore, these educational differences should be interpreted cautiously and should not be viewed as direct effects of education itself.

The regression models provided further insight into the independent associations of psychological and COVID-19-related variables with depressive symptoms and hopelessness. Emotional distress and impaired coping was the strongest positive predictor of EPDS scores in both waves and also the strongest positive predictor of BHS scores. This component captured a broad subjective distress domain rather than a single external stressor, including low mood, loss of pleasure, tension, sleep difficulties, concentration problems, perceived uselessness, and perceived inability to cope. Its strong association with both depressive symptoms and hopelessness suggests that the subjective emotional and cognitive experience of the pandemic context was closely linked to pregnant women’s mental health during both pandemic waves, consistent with previous evidence on pandemic-related uncertainty, disruption of prenatal care, fear of infection, and pregnancy-specific COVID-19 worries^7,22,30^.

Self-efficacy was a consistent negative predictor of both EPDS and BHS scores. Although the cross-sectional design does not allow conclusions about protective effects, the consistent inverse association suggests that lower perceived coping competence was linked to higher depressive symptoms and hopelessness. This finding is consistent with the role of self-efficacy as a coping-related psychological resource, reflecting perceived ability to manage challenging or stressful situations. In the context of pregnancy during a public health crisis, self-efficacy may be particularly relevant because women had to navigate uncertainty about healthcare access, infection risk, childbirth preparation, and family support while maintaining a sense of control and agency. Thus, the findings position self-efficacy as an important psychological correlate of mental health indicators in this population.

The predictors of EPDS scores differed partly between waves. In Wave 1, pregnancy-, childbirth-, and healthcare-related concerns and confinement-related difficulties were significant positive predictors, alongside emotional distress and impaired coping. This pattern may reflect the abrupt disruption of antenatal care expectations, hospital support, daily routines, and family arrangements during the early pandemic restrictions in Hungary. In Wave 2, hopelessness and financial and housing concerns were also significant positive predictors of EPDS scores. By this later stage, the higher epidemiological burden and renewed restrictions may have made practical and economic strain more salient for some women. These interpretations should remain cautious because the two samples were independent and the design was observational; the regression models indicate independent associations, not causal pathways.

## Limitations and strengths

Several limitations should be considered when interpreting the findings. First, the study used a repeated cross-sectional design based on two independent samples. Therefore, the results describe differences between pregnant women assessed during two distinct pandemic waves and do not allow conclusions about within-person change, individual adaptation over time, or causal relationships. The observed associations should be interpreted as concurrent relationships within each wave.

Second, data were collected using an anonymous online survey. This approach allowed rapid data collection during an evolving public health crisis, but it may also have introduced self-selection bias and limited participation among women with restricted internet access or lower digital literacy. The sample was not representative of all pregnant women in Hungary, as participants were predominantly married or living with a partner and a relatively high proportion had higher education. These characteristics may limit the generalizability of the findings, particularly to socially disadvantaged or otherwise vulnerable groups.

Third, all psychological variables were assessed using self-report questionnaires. Although the EPDS, BHS, and GSE scale are established psychological measures, self-report instruments cannot replace clinical interviews or diagnostic assessment. Accordingly, the EPDS categories used in the present study should be interpreted as screening-based symptom severity categories rather than clinical diagnoses.

Fourth, the study-specific „Pregnancy during the coronavirus” questionnaire was developed for the present research and was not a previously validated instrument. This was partly due to the rapidly evolving pandemic context and the limited availability of directly relevant pregnancy-specific COVID-19 stressor measures at the time of questionnaire development. Although the questionnaire was informed by professional expertise and pregnant women’s reported concerns, and its dimensionality and internal consistency were examined using PCA and Cronbach’s alpha, the component structure should be considered exploratory. Future studies should further develop and validate pregnancy-specific crisis-related stressor measures in independent samples, including instruments that can capture stressors arising during public health emergencies or other large-scale crises affecting antenatal care, social support, family functioning, and perceived safety during pregnancy.

Fifth, the Wave 1 and Wave 2 versions of the COVID-19 questionnaire were not fully identical. The Wave 2 version was adapted to reflect the changed pandemic context and included six additional items. To improve comparability, PCA was conducted on the common, non-conditional item pool, and direct cross-wave interpretations were restricted to overlapping content areas or conceptually comparable domains. Nevertheless, the modification of the questionnaire between waves should be considered when interpreting wave comparisons.

The study did not include a non-pregnant comparison group; therefore, the findings cannot determine whether the observed psychological patterns were specific to pregnancy or reflected broader pandemic-related distress among women of reproductive age. Future studies including non-pregnant comparison groups would be valuable for separating pregnancy-specific vulnerability from more general pandemic-related mental health effects.

Another limitation is that the study relied on self-report survey data and did not include objective medical or clinical indicators from medical records. Variables such as blood pressure, pre-pregnancy BMI, obesity, pregnancy complications, or other clinically verified health indicators were not available. During the pandemic, routine antenatal care was partly reorganized, and many contacts between pregnant women and health visitors or healthcare providers took place remotely. Therefore, collecting standardized objective clinical indicators within an anonymous online survey was not feasible. Future studies should combine self-report psychological data with medical records or routinely collected antenatal care data to better understand the interaction between physical health, pregnancy-related clinical factors, and mental health during public health crises.

Despite these limitations, the study has several strengths. It included a large sample of pregnant women assessed during real pandemic periods in Hungary, rather than relying on retrospective accounts collected after the acute phases of the crisis. The two-wave repeated cross-sectional design made it possible to compare psychological indicators and pregnancy- and COVID-19-related stressor domains across two distinct national pandemic contexts. The study also combined established psychological instruments with a pregnancy-specific COVID-19 questionnaire designed to capture concerns that were not covered by standard scales. This provides a broader view of how depressive symptoms, hopelessness, self-efficacy, and pandemic-related concerns were interrelated among pregnant women during the COVID-19 pandemic. To our knowledge, this is among the first large-sample Hungarian studies to examine mental health indicators and pregnancy-specific COVID-19-related concerns among pregnant women during real pandemic-wave periods.

## Implications for practice

The findings suggest that antenatal mental health screening during public health crises should not be limited to depressive symptoms alone. Although EPDS-based screening remains important, the present results indicate that hopelessness, perceived coping competence, pregnancy-specific uncertainty, healthcare-related concerns, and material stressors may also provide clinically relevant information. A broader screening approach could help identify pregnant women whose distress is not fully captured by depressive symptom scores, particularly those reporting emotional distress and impaired coping, low self-efficacy, unplanned or unwanted pregnancy, financial or housing concerns, or limited perceived support.

The strong associations of emotional distress and impaired coping with both depressive symptoms and hopelessness suggest that crisis-related antenatal support should include attention to emotion regulation, perceived control, and future-oriented worries. Cognitive-behavioural techniques may be useful for addressing catastrophic expectations, hopeless thinking, and perceived loss of control related to pregnancy, childbirth, infection risk, healthcare access, and family safety. Mindfulness-based strategies, pregnancy-adapted relaxation, breathing exercises, and brief stress-regulation techniques may also support women in managing uncertainty, heightened arousal, and sleep or concentration difficulties^41^. These approaches should be understood as supportive elements of care and not as substitutes for specialist mental health treatment when clinically significant symptoms are present.

The findings also underline the importance of clear and predictable communication in antenatal care during rapidly changing public health situations. Pregnant women may benefit from regularly updated information about antenatal visits, hospital procedures, support-person policies, infection-control measures, and pathways for urgent help^7,40^. Strengthening self-efficacy may therefore require not only psychological support, but also practical predictability: knowing whom to contact, what to expect if regulations change, and what forms of support are available. Because financial and housing concerns were relevant in the second wave, mental health assessment should also include social and material stressors. Questions about employment insecurity, housing difficulties, access to childcare, existing caregiving responsibilities, and reduced informal support may help professionals identify women whose psychological burden is shaped by broader social and economic pressures. In such cases, referral pathways should include not only psychological services, but also social work, family support services, and community-based resources.

In the Hungarian context, the health-visitor network may be especially important for early identification, psychoeducation, and referral, including when contact with pregnant women takes place partly remotely. Collaboration between health visitors, obstetric providers, midwives, psychologists, primary care professionals, and social services could help ensure that depressive symptoms, hopelessness, reduced coping capacity, pregnancy-specific fears, and social vulnerability are recognized early and addressed in an integrated way. Clinicians should also remain attentive to differential diagnosis when severe fear, perceived threat, loss of control, isolation, or distressing healthcare experiences are present. Although PTSD was not assessed in this study, trauma-related symptoms, acute stress reactions, anxiety-related distress, depressive symptoms, and hopelessness may require different clinical responses. Couple- or family-focused support may also be relevant when pandemic-related restrictions, caregiving demands, remote schooling, or reduced social contact place additional strain on family functioning and partner communication.

## Conclusion

The present study examined depressive symptoms, hopelessness, self-efficacy, and pregnancy- and COVID-19-related stressors among pregnant women in Hungary during two independent pandemic-wave samples. Depressive symptom and hopelessness categories did not differ significantly between the two waves, indicating that a clinically relevant psychological burden was present across distinct pandemic contexts. At the same time, the pattern of COVID-19-related stressors differed: confinement-related difficulties and daily disruption, as well as perceived support and positive reappraisal, were higher in Wave 1. Emotional distress and impaired coping was the strongest predictor of both depressive symptoms and hopelessness, while self-efficacy was consistently associated with lower symptom levels. In Wave 2, unplanned or unwanted pregnancy and financial and housing concerns were also relevant markers of psychological vulnerability. These findings underline the importance of pregnancy-sensitive mental health screening during public health crises, including attention to depressive symptoms, hopelessness, perceived coping capacity, pregnancy-specific uncertainty, and material stressors. Future longitudinal studies using validated crisis-specific instruments are needed to clarify how pandemic-related and broader public health stressors shape maternal mental health during pregnancy.

## Data Availability

The datasets generated and analysed during the current study are not publicly available. The informed consent provided to participants stated that the data would be accessible only to the research team and would be used anonymously and in aggregated form for statistical analysis. In addition, the dataset contains sensitive psychological and pregnancy-related information from pregnant women. Therefore, individual-level data cannot be shared publicly. Aggregated data supporting the findings of this study may be made available from the corresponding author upon reasonable request, in accordance with ethical and data protection requirements.
